# A Review of Mechanisms on the Beneficial Effect of Exercise on Atherosclerosis

**DOI:** 10.7759/cureus.11641

**Published:** 2020-11-23

**Authors:** Daniel Chacon, Brian Fiani

**Affiliations:** 1 Medicine, Ross University School of Medicine, Bridgetown, BRB; 2 Neurosurgery, Desert Regional Medical Center, Palm Springs, USA

**Keywords:** atherosclerosis, cardiovascular disease, coronary artery disease, exercise

## Abstract

Cardiovascular disease has affected a large percentage of the world, and as a result, we have had major advancements in pharmacological and procedural intervention of this disease. With the increased burden of rising healthcare costs, alternative treatment with exercise has shown to be much more cost effective and just as beneficial to patients compared to pharmacological and procedural treatment. We highlight some of the major mechanisms behind the beneficial effect of exercise on atherosclerosis and hope to encourage patients and providers to attempt to adopt this form of treatment that has not only shown to be beneficial to heart disease, but diseases such as diabetes and obesity as well.

## Introduction and background

As the leading cause of mortality in the USA [1], cardiovascular disease has arguably become the standard to gauge an individual’s health. It is estimated that 610,000 people per year in the USA die of heart disease [2]. In the latest report by the American Heart Association, 365,914 Americans in 2017 died specifically due to coronary heart disease [3], otherwise known as atherosclerosis. Largely due to chronic inflammation in the coronary arteries, atherosclerosis results from the formation of an atheroma with the potential for luminal occlusion, resulting in decreased blood flow to the myocardium [4]. Such decreases in blood flow can result in angina and in some cases, a myocardial infarction [5].

With such a high prevalence of heart disease in the population, pharmacological and procedural intervention have taken the forefront of treatment. Medical science has proven the efficacy of pharmacological treatment such as statins for hyperlipidemia and ace inhibitors, beta blockers and diuretics for hypertension; meanwhile, procedures such as angioplasty have shown to improve symptoms of angina in patients with coronary heart disease. Published papers and clinical trials have shown through the explanation of mechanisms how exactly these treatments are proven to work. In a no-contest explanation of inhibiting 3-hydroxy-3-methylglutaryl coenzyme A (HMG-CoA) reductase or the renin-angiotensin system, we accept the pharmacological treatment of heart disease, while angiographs demonstrate the efficacy of stents after percutaneous coronary intervention. As healthcare costs continue to rise, a once gold standard treatment needs to be prescribed just as often, and that is exercise.

Ongoing research has made the effort of identifying mechanisms behind the beneficial effect of exercise on heart disease, specifically atherosclerosis. In hopes of returning exercise to its gold standard for treatment of heart disease, we will discuss current proposed mechanisms of the beneficial effects on the heart, as it relates to atherosclerosis and the coronary arteries. Can exercise decrease mortality after a heart attack? Is it possible to reverse plaque buildup in the coronary arteries? We will entertain these questions and others throughout this paper.

## Review

Atherosclerosis: a brief overview of pathophysiology

A high serum low-density lipoprotein (LDL) cholesterol level is a known risk factor for atherosclerosis and is the element responsible for initiating the pathogenesis of the disease [2,6-8]. High circulating levels of LDL lead to an increased opportunity for accumulation in the arterial intima [6]. LDL particles diffuse through the endothelial cells lining the vessels where they will undergo modification such as oxidation, lipolysis, and proteolysis and cause inflammation [2,6]. This attracts large numbers of macrophages and lymphocytes to the site of accumulation and the modified LDL is taken up for degradation resulting in the formation of foam cells which make up the core of the atherosclerotic lesion [2,6]. Smooth muscle cells called on by cytokines secreted by macrophages migrate to the area of the lesion and produce an extracellular matrix surrounding the core with a fibrous cap [2,6]. The cap surrounding the atheroma is vulnerable to erosion by enzymes such as matrix metalloproteinase and myeloperoxidase which provide the potential for rupture and exposure of the core contents and activation of the coagulation cascade resulting in a thrombus [2,6]. As the process repeats over years, the atheroma grows in size with the potential for partial and total occlusion of the coronary artery [8]. Reducing blood flow to the myocardium manifests as angina during periods of increased oxygen demand by the heart [9]. A myocardial infarction may result from a plaque rupture setting off the coagulation cascade resulting in total occlusion of the vessel [2,6]. 

Proposed mechanisms on the effect of exercise on atherosclerosis

The effect of exercise on general health has largely been accepted as beneficial on issues such as blood pressure, insulin sensitivity, and weight loss [10]. Meanwhile, ongoing research has made attempts at identifying the mechanisms behind the beneficial effect of exercise on coronary heart disease [11]. Several mechanisms such as cardiac preconditioning, regression of plaque formation, and increased coronary collateralization are a few of the many that have been proposed as explanations for these effects [11-12]. As more research is conducted, we will be able to better understand the relationship between exercise and coronary heart disease. Table 1 provides a summary of the beneficial effects of exercise and their proposed mechanisms that will be discussed. 

**Table 1:** Summary of the Beneficial Effects of Exercise and Proposed Mechanisms ROS: reactive oxygen species.

Beneficial Effect	Proposed Mechanism
Cardiac Pre-conditioning	Increased ROS Scavenging
Increased Collateralization	Arteriogenesis
Plaque Regression	Increased High Density Lipoprotein, Macrophage/Foam Cell Migration

Cardiac preconditioning

As a potentially fatal outcome of atherosclerosis, protection against myocardial infarction has been an attractive target in cardiology research. Although more research is needed, mechanisms linked to exercise have been discovered that have the potential to decrease mortality after an ischemic/reperfusion (I/R) injury due to coronary events [12-15]. 

Cardiac preconditioning in exercised individuals has shown magnificent potential in decreasing the pathologic process after I/R injury [12]. Research has shown a positive correlation between exercised individuals and survival of a myocardial infarction, with the ability to reduce infarct size and delay injurious effects from I/R injury [12-15]. It has been proposed that a delay in damage and cell death due to metabolic change during a myocardial infarction could account for this benefit [12]. As much as a 30%-40% reduction in damage due to infarct has been attributed to the beneficial effect of exercise cardiac preconditioning [12]. The major mechanism involved in these cardioprotective effects is the increased ability to clear reactive oxygen species (ROS) from the injured tissue.

Increased ROS Scavenging

A major component of cardiac preconditioning is the enhanced ability to clear ROS due to I/R injury [12-15]. Increases in compounds such as nicotinamide adenine dinucleotide phosphate (NADPH) oxidase, superoxide, and xanthine oxidase have been shown to damage heart tissue [12]. Exercise has been shown to increase ROS during physical activity, leading to the notion that exercise may possibly influence cardiac adaptations that result in protection against infarction through increased clearance of ROS [12,13,15]. 

Superoxide dismutase (a known enzyme that catalyzes ROS), specifically manganese superoxide dismutase (MnSOD) mainly found in the mitochondria has been shown to increase after exercise and has also been positively correlated with protection from infarction [12-15]. This has been observed in studies where regular exercise over several days has shown increases in MnSOD and has shown to retain the cardioprotective effect in exercised older hearts [12]. In genetic experiments with mice, overexpression and MnSOD resulted in an increase of cardioprotective effects against infarction, meanwhile under expression of MnSOD resulted in heart tissue damage from I/R injury due to decreased cardiac function [12-13,16-17]. While cardiac preconditioning and its mechanism of enhanced ROS clearance show potential benefits on heart disease, more research needs to be done to further solidify its contribution to the beneficial effect of exercise on atherosclerosis.

Increased coronary collateralization

Enhanced coronary collateralization has shown promise as a potential mechanism for the beneficial effects of exercise on atherosclerosis [18]. Each coronary artery supplies blood to its respective area of myocardium as it branches off the aorta. In the case of a stenotic vessel due to atherosclerosis, the area of the myocardium distal to the occlusion is subject to ischemia due to decreased blood supply contingent upon the degree of stenosis, and in certain cases, may result in an infarction due to total occlusion. Coronary collateralization involves arterioles that have the ability to provide relief from decreased blood supply and may decrease infarct size due to an increase in retrograde blood flow to the ischemic myocardium [18-20]. Studies examining the effects of exercise on coronary collateralization have shown mechanisms including increased ischemia due to stenotic vessels and increases in fluid shear stress influence the utilization and development of collateralization to an area of ischemic myocardium [18-21]. 

An important aspect is the collateral flow index (CFI), which is a measurement of the amount of blood flow in a stenosed coronary artery, that can be attributed to collateral circulation calculated as - (mean distal coronary pressure - central venous pressure) / (mean aortic pressure - central venous pressure) [22]. Much of the assessments of collateralization have taken place in the presence of coronary artery disease, and the intent of these studies is to explore the possibility of coronary collateralization supplying blood to ischemic myocardial tissue in the case of coronary stenosis or myocardial infarction. It has been shown that a collateral blood flow calculated as a CFI of at least .20-.25 contributed to absent signs of myocardial ischemia on ECG when testing for CFI itself [19,22-23]. This has the potential to explain the importance of enhanced collateralization as it pertains to cardioprotective effects during ischemia. A similar result was shown in a separate study where patients without coronary stenosis that had functional collateralization did not show symptoms of myocardial ischemia during vascular occlusions [19,24], further suggesting that an increased coronary collateral circulation may be a worthy mechanism of exploring.

Exercise has shown to increase the ability of collateral vessels to supply blood to tissue downstream from stenosis through the measurement of the CFI. It has been observed that regular exercise in the presence of coronary artery disease has shown to enhance the development of coronary collateralization through the promotion of arteriogenesis, which is the necessary type of development to increase blood flow to collateral dependent areas of the myocardium [19,20]. This was the case in a report by Zbinden et al, where they show that CFI was significantly increased in not only stenotic vessels, but non-stenotic vessels after completion of an exercise program, meanwhile this was not shown in the control group who did not undergo exercise [20,25]. In a separate study by Belardinelli et al, they report that after eight weeks of exercise training in patients with coronary artery disease, the exercised individuals exhibited an angiographically proven increase in collateralization formation that resulted in increased perfusion and improved myocardial contractility [20,26]. Furthermore, Seiler et al. reported that increased physical activity in patients with coronary artery disease resulted directly and indirectly in collateral blood flow to ischemic areas of myocardium following occlusion [20-21]. 

Arteriogenesis

Arteriogenesis is the process in which coronary collateralization has the capability of salvaging ischemic myocardium due to stenosis or infarction [19,27,28]. In patients with coronary artery disease, bouts of ischemia whether due to high-grade stenosis or induced by exercise, have a positive effect on collateral development and utilization [19,20,29]. During exercise in a patient with coronary artery disease, the stenotic artery causes a decrease in blood flow distal to the occlusion and leads to an increase in redirected blood flow to the collateral vessels [18-19,29]. As pressure drops in the occluded vessel, blood flows retrograde through the collateral circulation providing oxygen to ischemic tissue, and the increase in fluid shear stress causes an increase in endothelial factors leading to arteriogenesis [19-20,29-32]. 

Arteriogenesis leads to growth in collateral vessel length and diameter mediated by an increase in endothelial cell factors due to increases in fluid shear stress [32]. The release of monocyte chemoattractant protein-1 (MCP-1), tumor necrosis factor-alpha (TNF-alpha), and beta-fibroblast growth factor (b-FGF) by endothelial cells along with the migration of smooth muscle cells and monocytes leads to the morphological changes seen in this process [32]. Increased release of matrix metalloproteinase (MMP) by endothelial cells leads to degradation of the lamina elastica interna in the arteriogenic vessel [32-34]. As the vessel undergoes transformation, smooth muscle cells recruited to the area by b-FGF produce elastin that replace the lamina elastica interna after its degradation, and the new expanded lumen and is now able to accommodate the increases in collateral blood flow [32]. This process was shown to be beneficial as an inverse relationship was shown between the number of collateral vessels and increased collateral vessel luminal diameter [32]. Suggesting that the enhanced ability of collateral flow is not due to an increase in the number of new vessels, but to the increase in luminal diameter induced by the increase of blood flow to these vessels by coronary artery stenosis [32]. As an attractive mechanism for the beneficial effect of exercise on atherosclerosis, arteriogenesis shows promise as a possible explanation for the cardioprotective effects of exercise. 

Plaque regression

As plaque continues to accumulate, as does the likelihood of severe coronary stenosis and myocardial infarction. A popular area of research on the beneficial effects of exercise on atherosclerosis has been focused on the possibility of plaque regression. Studies have been conducted focusing specifically on the ability of exercise to not only halt the progression of atherosclerosis, but also the regression of existing plaques. Promising results by Okabe et al. showed that fatty streak formation was suppressed in Apo-E deficient mice fed a high-fat diet after eight or sixteen weeks of exercise compared to the non-exercised control group [34]. Upon tissue processing by oil red O staining, the control mice were shown to have a larger plaque size compared to the exercised mice [34]. Adding to the notion that exercise may have a role in suppressing plaque formation.

In humans, the Lifestyle Heart Trial studied the effect of lifestyle changes in patients with coronary artery disease which included three hours of exercise per week [35-36]. The trial showed that after one year of exercise in the experimental group, patients not only showed a decrease in the frequency and severity of symptoms of angina but also a regression of plaque size in existing coronary atherosclerotic lesions [35-36]. This is in contrast to the control group which showed progression of existing coronary atherosclerotic lesions after the trial [35-36]. In another study analyzing the effect of exercise effect on atherosclerosis, the Heidelberg regression study by Hambrecht et al. found that regression in coronary lesions was observed in patients that burned greater than 2200 calories per week, which equals to about 5-6 hours of exercise per week [36-37]. Furthermore, in a study by Tani et al, a 12.9% regression of plaque size was observed in patients after six months of lifestyle modification that included exercise along with statin therapy and diet modification [36,38]. Mechanisms such as increased high-density lipoprotein (HDL) and lowered LDL, along with clearing of macrophages and foam cells from the necrotic core of the lesion have been discovered as a potential mechanism explaining the beneficial effect of exercise on plaque regression [39]. 

Increased High-Density Lipoprotein

As an accepted risk factor for atherosclerosis, a high LDL cholesterol has shown to be the beginning process of the pathophysiology of the disease [2,6-8]. Meanwhile, serum levels of HDL cholesterol have been inversely correlated with coronary artery disease [39-40]. It has also been established that increased levels of HDL have been accepted as participating in the process of plaque regression [39]. Reverse cholesterol transport (RCT) by HDL plays a major role in the process and although controversial among researchers, it is accepted that the mechanism behind HDL’s beneficial effect on plaque regression is that of RCT of peripheral cholesterol back to the liver [39]. Macrophages filled with oxidized LDL called foam cells make up the necrotic core of the atherosclerotic lesion, and as the core continues to fill while being caped by smooth muscle cells, the size of the lesion and degree of coronary stenosis increases [2,6]. HDL works by reverse transporting LDL cholesterol from the necrotic core and macrophages found in the coronary artery to the liver [39]. There are four mechanisms of uptake of cholesterol from the periphery that represent the beginning process of RCT [39]. The types of cholesterol uptake by HDL include aqueous diffusion, scavenger receptor class B type 1 (SR-BI) mediated efflux, adenosine triphosphate (ATP) binding cassette transporter A1 (ABC) mediated efflux, and ABCG1 mediated efflux [39]. These processes retrieve cholesterol from peripheral stores and transport them back to hepatocytes in the liver; the excess cholesterol is ultimately excreted through the hepatobiliary route into the intestinal lumen where RCT is considered complete [39]. 

It has been shown that exercise has a positive effect on increasing levels of serum HDL [41-43]. In a meta-analysis concentrating on studies observing the effect of exercise on HDL levels, they report that regular aerobic exercise was shown to increase levels of HDL contingent upon a minimum energy expenditure of 900 calories per week [43]. In a separate meta-analysis of randomized controlled trials, it was concluded once again that exercise showed a statistically significant increase in HDL by 11% in the exercised groups [44]. It was also shown that exercise not only has a positive effect on HLD, but LDL and triglycerides levels as well [45]. A study reviewing the effect of exercise on total blood lipid profile showed that after 12 weeks of an exercise program, HDL levels increased by an average of 4.6%, while LDL and triglycerides decreased by 5% and 3.7%, respectively [45-46]. We encourage the reader to review a report by Mann et al. where they conduct yet another meta-analysis of studies on the effect of exercise on HDL levels that report many more conclusions that HDL levels are indeed positively influenced by exercise [45]. 

Macrophage/Foam Cell Migration

Another contributing factor to plaque regression is macrophage and foam cell migration from the necrotic core of the lesion back to lymph nodes [39,47]. Previously believed that apoptosis played the sole role in macrophage disappearance from the core, it is now known that a regain in motility is involved as well [39]. It has been observed that the mechanism behind the returned motility includes growth of the macrophage lamellipodia for movement [39], however, the process preceding this new ability is extensively tedious in nature and is beyond the scope of discussion for this paper.

## Conclusions

Physical inactivity combined with poor diet leading to increased levels of LDL cholesterol have shown to be risk factors for developing cardiovascular disease. The use of pharmacological and procedural intervention has become common practice to treat heart diseased patients as it has been proven to work after much research. However, it is important to consider other factors that might accompany the patient such as socioeconomic status. Exercise has been largely accepted as beneficial to all patients, especially those with heart disease; not only has this alternative treatment shown to be beneficial but cost effective as well. Mechanisms such as cardiac preconditioning, increased coronary collateralization, and plaque regression are few of the many mechanisms that have been shown to potentially explain the benefits of exercise on the heart. This is an important avenue of research and discussion as heart disease has affected populations around the world. Although the aforementioned mechanisms have gained popularity among clinical researchers, much more work needs to be done for these explanations to be widely accepted as truth and allow them to move to the forefront of treatment for heart disease.

## References

[1] D'Souza MJ, Li RC, Gannon ML, Wentzien DE (2019). 1997-2017 leading causes of death information due to diabetes, neoplasms, and diseases of the circulatory system, issues cautionary weight-related lesson to the US population at large. https://ieeexplore.ieee.org/abstract/document/8863033.

[2] Pahwa R, Jialal I (2020). Atherosclerosis. https://www.ncbi.nlm.nih.gov/books/NBK507799/?report=classic.

[3] Virani SS, Alonso A, Benjamin EJ (2020). Heart disease and stroke statistics-2020 update: a report from the American Heart Association. Circulation.

[4] Rafieian-Kopaei M, Setorki M, Doudi M, Baradaran A, Nasri H (2014). Atherosclerosis: process, indicators, risk factors and new hopes. Int J Prev Med.

[5] Gillen C, Goyal A (2020). Stable Angina. https://www.ncbi.nlm.nih.gov/books/NBK559016/.

[6] Lusis AJ (2000). Atherosclerosis. Nature.

[7] Al-Mamari A (2009). Atherosclerosis and physical activity. Oman Med J.

[8] Herrington W, Lacey B, Sherliker P, Armitage J, Lewington S (2016). Epidemiology of atherosclerosis and the potential to reduce the global burden of atherothrombotic disease. Circ Res.

[9] Hermiz C, Sedhai YR (2020). Angina. https://www.ncbi.nlm.nih.gov/books/NBK557672/.

[10] Nystoriak MA, Bhatnagar A (2018). Cardiovascular effects and benefits of exercise. Front Cardiovasc Med.

[11] Bowles DK, Laughlin MH (2011). Mechanism of beneficial effects of physical activity on atherosclerosis and coronary heart disease. J Appl Physiol.

[12] Frasier CR, Moore RL, Brown DA (2011). Exercise-induced cardiac preconditioning: how exercise protects your achy-breaky heart. J Appl Physiol.

[13] Quindry JC, Hamilton KL (2013). Exercise and cardiac preconditioning against ischemia reperfusion injury. Curr Cardiol Rev.

[14] Chowdhury MA, Sholl HK, Sharrett MS, Haller ST, Cooper CC, Gupta R, Liu LC (2019). Exercise and cardioprotection: a natural defense against lethal myocardial ischemia-reperfusion injury and potential guide to cardiovascular prophylaxis. J Cardiovasc Pharmacol Ther.

[15] Marongiu E, Crisafulli A (2014). Cardioprotection acquired through exercise: the role of ischemic preconditioning. Curr Cardiol Rev.

[16] Loch T, Vakhrusheva O, Piotrowska I, Ziolkowski W, Ebelt H, Braun T, Boner E (2009). Different extent of cardiac malfunction and resistance to oxidative stress in heterozygous and homozygous manganese-dependent superoxide dismutase-mutant mice. Cardiovasc Res.

[17] Clark BJ III, Acker MA, McCully K (1988). In vivo 31P-NMR spectroscopy of chronically stimulated canine skeletal muscle. Am J Physiol.

[18] Laughlin MH, Bowles DK, Duncker DJ (2012). The coronary circulation in exercise training. Am J Physiol Heart Circ Physiol.

[19] Bigler MR, Seiler C (2019). The human coronary collateral circulation, its extracardiac anastomoses and their therapeutic promotion. Int J Mol Sci.

[20] Heaps CL, Parker JL (2011). Effects of exercise training on coronary collateralization and control of collateral resistance. J Appl Physiol.

[21] Senti S, Fleisch M, Billinger M, Meier B, Seiler C (1998). Long-term physical exercise and quantitatively assessed human coronary collateral circulation. J Am Coll Cardiol.

[22] de Marchi SF, Streuli S, Haefeli P (2012). Determinants of prognostically relevant intracoronary electrocardiogram ST-segment shift during coronary balloon occlusion. Am J Cardiol.

[23] Traupe T, Gloekler S, de Marchi SF, Werner GS, Seiler C (2010). Assessment of the human coronary collateral circulation. Circulation.

[24] Wustmann K, Zbinden S, Windecker S, Meier B, Seiler C (2003). Is there functional collateral flow during vascular occlusion in angiographically normal coronary arteries?. Circulation.

[25] Zbinden R, Zbinden S, Meier P (2007). Coronary collateral flow in response to endurance exercise training. Eur J Cardiovasc Prev Rehabil.

[26] Belardinelli R, Georgiou D, Ginzton L, Cianci G, Purcaro A (1998). Effects of moderate exercise training on thallium uptake and contractile response to low-dose dobutamine of dysfunctional myocardium in patients with ischemic cardiomyopathy. Circulation.

[27] Arras M, Ito WD, Scholz D, Winkler B, Schaper J, Schaper W (1998). Monocyte activation in angiogenesis and collateral growth in the rabbit hindlimb. J Clin Invest.

[28] Seiler C, Pohl T, Wustmann K (2001). Promotion of collateral growth by granulocyte-macrophage colony-stimulating factor in patients with coronary artery disease: a randomized, double-blind, placebo-controlled study. Circulation.

[29] Prior BM, Lloyd PG, Yang HT, Terjung RL (2003). Exercise-induced vascular remodeling. Exerc Sport Sci Rev.

[30] Gloekler S, Meier P, de Marchi SF (2010). Coronary collateral growth by external counterpulsation: a randomised controlled trial. Heart.

[31] Pipp F, Boehm S, Cai WJ (2004). Elevated fluid shear stress enhances postocclusive collateral artery growth and gene expression in the pig hind limb. Arterioscler Thromb Vasc Biol.

[32] van Royen N, Piek JJ, Buschmann I, Hoefer I, Voskuil M, Schaper W (2001). Stimulation of arteriogenesis; a new concept for the treatment of arterial occlusive disease. Cardiovasc Res.

[33] Taraboletti G, D'Ascenzo S, Borsotti P, Giavazzi R, Pavan A, Dolo V (2002). Shedding of the matrix metalloproteinases MMP-2, MMP-9, and MT1-MMP as membrane vesicle-associated components by endothelial cells. Am J Pathol.

[34] Okabe TA, Kishimoto C, Murayama T, Yokode M, Kita T (2006). Effects of exercise on the development of atherosclerosis in apolipoprotein E-deficient mice. Exp Clin Cardiol.

[35] Ornish D, Brown SE, Scherwitz LW (1990). Can lifestyle changes reverse coronary heart disease?: The Lifestyle Heart Trial. Lancet.

[36] Winzer EB, Woitek F, Linke A (2018). Physical activity in the prevention and treatment of coronary artery disease. J Am Heart Assoc.

[37] Hambrecht R, Niebauer J, Marburger C (2010). Various intensities of leisure time physical activity in patients with coronary artery disease: effects on cardiorespiratory fitness and progression of coronary atherosclerotic lesions. J Am Coll Cardiol.

[38] Tani S, Nagao K, Anazawa T (2010). Coronary plaque regression and lifestyle modification in patients treated with pravastatin. Circ J.

[39] Francis AA, Pierce GN (2011). An integrated approach for the mechanisms responsible for atherosclerotic plaque regression. Exp Clin Cardiol.

[40] Gordon T, Castelli WP, Hjortland MC, Kannel WB, Dawber TR (1977). High density lipoprotein as a protective factor against coronary heart disease: the Framingham Study. Am J Med.

[41] Ruiz-Ramie JJ, Barber JL, Sarzynski MA (2019). Effects of exercise on HDL functionality. Curr Opin Lipidol.

[42] Durstine JL, Grandjean PW, Davis PG, Ferguson MA, Alderson NL, DuBose KD (2001). Blood lipid and lipoprotein adaptations to exercise: a quantitative analysis. Sports Med.

[43] Kodama S, Tanaka S, Saito K (2007). Effect of aerobic exercise training on serum levels of high-density lipoprotein cholesterol: a meta-analysis. Arch Intern Med.

[44] Kelley GA, Kelley KS (2006). Aerobic exercise and HDL2-C: a meta-analysis of randomized controlled trials. Atherosclerosis.

[45] Mann S, Beedie C, Jimenez A (2013). Differential effects of aerobic exercise, resistance training and combined exercise modalities on cholesterol and the lipid profile: review, synthesis and recommendations. Sports Med.

[46] Leon AS, Sanchez OA (2001). Response of blood lipids to exercise training alone or combined with dietary intervention. Med Sci Sports Exerc.

[47] Clair RWS (1983). Atherosclerosis regression in animal models: current concepts of cellular and biochemical mechanisms. Prog Cardiovasc Dis.

